# Repeatability of a multi-segment foot model with a 15-marker set in healthy adults

**DOI:** 10.1186/1757-1146-7-24

**Published:** 2014-04-22

**Authors:** Sang Gyo Seo, Dong Yeon Lee, Hyuk Ju Moon, Sung Ju Kim, Jihyeung Kim, Kyoung Min Lee, Chin Youb Chung, In Ho Choi

**Affiliations:** 1 Department of Orthopedic Surgery, Seoul National University Hospital, 101 Daehak-no, Jongno-gu, Seoul 110-744, South Korea; 2 Department of Orthopedic Surgery, Seoul Metropolitan Government Seoul National, Korea University Boramae Medical Center, 20 Boramae-ro 5-gil, Dongjak-gu, Seoul 156-707, South Korea; 3 Department of Orthopaedic Surgery, Seoul National University Bundang Hospital, 300 Gumi-Dong, Bundang-Gu, Sungnam, Kyungki 463-707, South Korea

**Keywords:** Gait analysis, Repeatability, Multi-segment foot model, Foot 3D

## Abstract

**Background:**

Several 3D multi-segment foot models (MFMs) have been introduced for the in vivo analysis of dynamic foot kinematics. However, reproducibility of a model should be checked and ascertained before clinical utilization of a MFM. The purpose of this study was to determine the reliability of recently introduced MFM with a 15-marker set by assessing the participant’s stride-to-stride (intra-session) and visit-to-revisit (inter-session) repeatability.

**Methods:**

Twenty healthy adults with a mean age of 28.9 years (10 males and 10 females) were tested. Three representative strides from five separate trials were used for analysis from each session. Kinematic data of foot segmental motion was collected and tracked using the Foot3D Multi-Segment Software (Motion Analysis Co., Santa Rosa. CA). A retest was performed in the same manner at an interval of 4 weeks. Coefficients of multiple correlation (CMC) and intra-class correlation coefficient (ICC) were calculated in order to assess the intra-session and inter-session repeatability.

**Results:**

Inter-segment foot angles from healthy adults from a MFM with 15-marker set showed a narrow range of variability during the gait cycle. The mean intra-session ICC was 0.981 (±0.010), which was interpreted as excellent. The mean intra-session CMC was 0.948 (±0.027), which was interpreted as very good repeatability. The mean inter-session ICC was 0.886 (±0.047) and the mean inter-session CMC was 0.801 (±0.077), which were interpreted as excellent and good repeatability, respectively.

**Conclusion:**

We demonstrated a MFM with a 15-marker set had high intra-session and inter-session repeatability, especially in sagittal plane motion. We thought this MFM would be applicable to evaluation of the segmental foot motion during gait.

## Background

The characterization of foot mechanics during the gait cycle in healthy and diseased humans has been a challenge. In trials using three-dimensional (3D) analysis of opto-reflective markers, a ‘gold standard’ method to represent the actual motion of the tarsal bones might be the use of intra-cortical bone markers [1,2], although clinical application may be limited because of its invasiveness. A less invasive approach is to use skin mounted markers instead of bone markers to evaluate segmental foot motions. In the last two decades, several 3D multi-segment foot models (3D MFMs) have been introduced for the in vivo analysis of dynamic foot kinematics [3-12]. Although there are intrinsic weaknesses in each of these systems, such as skin motion artifact and reproducibility of marker location, 3D MFMs have potential benefits compared with a single-segment foot model gait analysis. In general, the reproducibility of 3D MFMs is thought to be classified as good [3,4,7,9,11-19]. There is also increasing evidence that the utilization of 3D MFMs in a clinical setting would enable physicians to assess functional disability and treatment outcome more objectively [20-25].

These models differ in the number of foot segments analyzed, the position of markers within each segment, and the mathematical interpretation of segmental motion, leading to different segmental motion patterns during gait cycle [26]. For example, there are varying numbers of markers placed around the foot and ankle even among thoroughly validated models: eleven markers in the Milwaukee Foot Model (MiFM) [7,27,28], 12 markers in the Heidelberg foot measurement method (HFM) [3], 13 markers in the Oxford Foot Model (OFM) [4,29,30], and 16 markers in the Leardini Foot Model (LFM) [9,10]. Ideally, increasing number of markers with accurate placement enables more precise analysis of the actual segmental foot motion. However, considering that the major source of variability in quantitative kinematic data is the difference of marker placement [28,31,32], precise standardization of marker placement is essential for proper interpretation of proposed MFMs.

Recently, Henley et al. proposed a 3D MFM of a 15-marker set with the goal of improving clinician’s ability to accurately implement the model in a clinical setting [8]. This model involves the placement of ten markers on prominent anatomical points around the foot and ankle with notable absence of medial and lateral calcaneal markers. Although a peer reviewed study utilizing this model has been recently published [33], it is difficult to judge the reliability of this model as it has yet to be reported in peer-reviewed journal.

The purpose of this study was to determine the reliability of a 3D MFM with a 15-marker set by assessing the participant’s stride-to-stride (intra-session) and visit-to-revisit (inter-session) repeatability.

## Methods

### Participants

This study was approved by the institutional review board of Seoul National University Hospital and all participants gave informed consents prior to participation. Twenty healthy adults aged 20–35 years were tested at the Laboratory of Human Motion Analysis in Seoul National University Hospital. Volunteers were recruited from the local area with equal numbers of males and females. Inclusion criteria were 1) no history of fracture or surgery on the lower extremities; 2) no subjective symptom during gait; 3) no abnormal findings on foot radiograph; 4) no history of cardiac or respiratory disease or uncorrected visual impairment; and 5) in normal function of the foot and ankle (AOFAS ankle-hindfoot score of 100). The alignment and range of motion of the lower extremity joints (the hip, knee and ankle) were clinically evaluated by authors (DYL, SGS) to exclude abnormal condition of the lower extremities. The mean age was 28.9 years (range 20-35) and the mean weight was 66.5 kg (range 44.9-105.5). The mean height was 168.5 cm (range 154.3-181.5) and the mean BMI was 23.3 kg/m^2^ (range 16.8-32.2). The mean spine-malleolar distance (SMD) was 86.0 cm (range 76.7-96.0). The mean foot length was 24.5 cm (range 21.5-26.9) and the mean foot width was 9.7 cm (range 8.6-11.0). Demographic data are presented in Table 1. Only one side was selected randomly for statistical analysis.

**Table 1:** Demographic data of participating subjects

	**Male (range)**	**Female (range)**
Demographic measurements		
Age (years)	29.2 (24-35)	27.8 (20-35)
Height (cm)	175.2 (168.3-181.5)	161.8 (154.3-165.5)
Weight (kg)	73.9 (60.3-105.5)	59.0 (44.9-78.4)
Body Mass Index (kg/m^2^)	24.0 (19.7-32.2)	22.5 (16.8-28.8)
Spine Malleolar Distance (cm)	88.1 (84.5-96.0)	81.4 (76.7-86.2)
Foot measurement		
Foot Length (cm)	25.6 (24.0-26.9)	23.4 (21.5-24.8)
Foot Width (cm)	10.1 (9.4-11.0)	9.3 (8.6-10.2)

### Marker set

When evaluating segmental foot motion, we used 15 opto-reflective markers on the anatomical landmarks of each knee, tibial shank, ankle and foot. This system consisted of 6 additional markers per foot in addition to the conventional Cleveland clinic lower extremity marker set [34]. Placement of the markers is described below.

**Figure 1:**
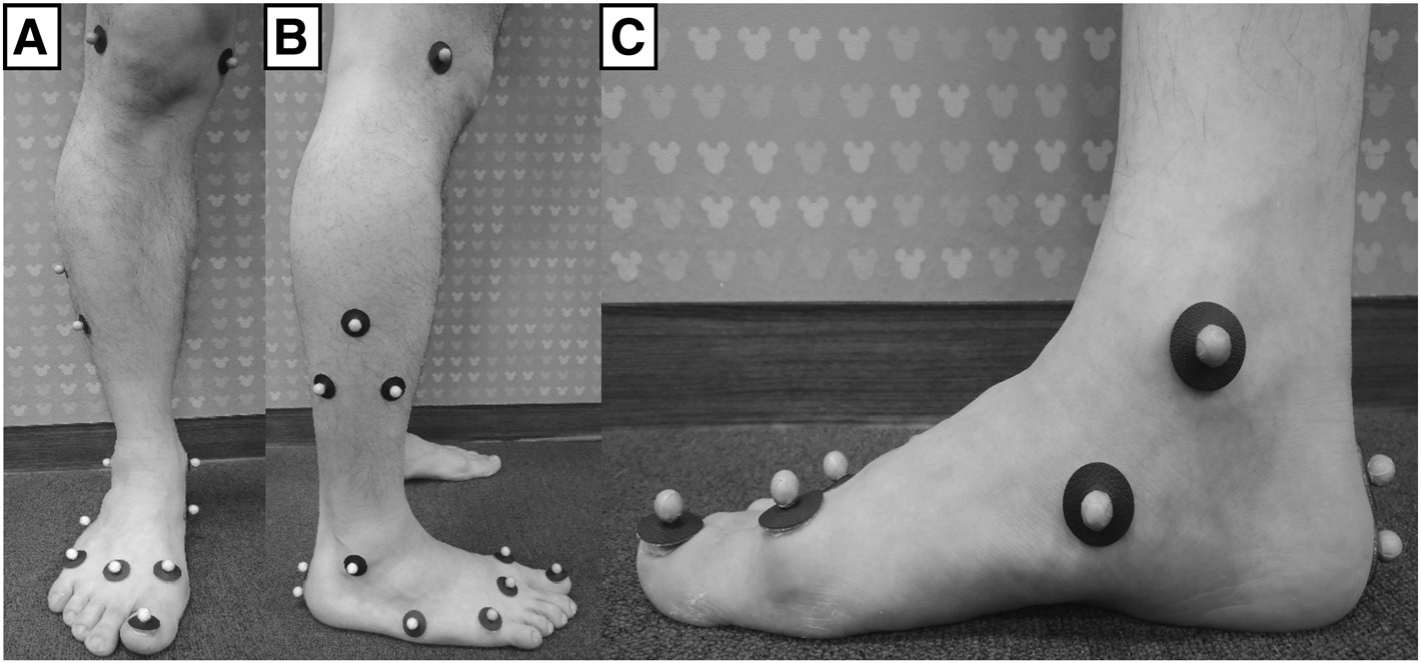
**Marker placement of a 3D multi-segment foot model with 15-marker set.** Ten markers were placed around the foot and ankle. **(A)**, **(B)** Anterior and lateral view of marker placement. **(C)** Hallux marker was placed in the middle of the hallux nail bed, 1st metatarsal marker on the dorsal metatarsal head just proximal to the 1st metatarsophalangeal joint, navicular marker on the most prominent point of the navicula, and two calcaneus markers were applied to the hindfoot.

Five markers were placed around the knee and tibial shank for calculation of the shank coordinate system. Four markers were placed on the ankle and hindfoot (one on the medial and lateral malleolus, and two on the calcaneus), 2 markers on the midfoot (navicular and cuboid) and 4 on the forefoot (three on the metatarsal area and one on the hallux) (Figure 1) [8]. More detailed description of marker position is presented in Table 2. All markers were placed by one operator experienced in the placement of the conventional Cleveland clinic lower extremity marker set, with reference to the standardized protocol with photography. Our gait laboratory has no previous experience with 3D MFMs.

**Table 2:** Marker placement of a multi-segment foot model with a 15-marker set

**Name of marker**	**Position of marker**
Knee Medial	Along the flexion/extension axis of rotation at medial femoral condyle
Knee Lateral	Along the flexion/extension axis of rotation at lateral femoral condyle
Shank Upper	Upper apex of the Shank triangle at the lateral aspect of middle lower leg
Shank Front	Lower front of the Shank triangle at the lateral aspect of middle lower leg
Shank Rear	Lower rear of the Shank triangle at the lateral aspect of middle lower leg
Ankle Medial	Apex of the medial malleolus
Ankle Lateral	Apex of the lateral malleolus
Heel	On the line bisecting posterior aspect of the heel at the height of the toe marker
Heel Distal	On the line bisecting posterior aspect of the heel below Heel marker and just above the fat pad
Navicula	The most prominent point of the navicula (navicular tuberosity)
Cuboid	Just proximal and superior to the base of the 5th metatarsal bone
MTH1	Dorsal metatarsal head just proximal to the 1st metatarsophalangeal joint
Toe	Dorsal web space just proximal between the 2nd and 3rd metatarsophalangeal joint
MTH5	Dorsal metatarsal head just proximal to the 5th metatarsophalangeal joint
Hallux	In the middle of the hallux nail bed

### Experimental procedures

After explaining the procedures and obtaining written consent, we measured each participant’s demographic data including height, body weight, leg length, foot length and width. The participants performed a 5-minute warm-up protocol walking comfortably. After warming up, the reflective markers were attached to foot and lower extremities of each participant. Participants were asked to walk at a comfortable speed along an 8 m walkway. We captured a static standing trial with the individual in anatomic position. After static calibration, kinematic gait data were collected using 12 cameras with a 3D optical motion capture system (OrthoTrak v6.6.4., Motion Analysis Co., Santa Rosa. CA) at a sample rate of 120 Hz. Eva Real-Time software (EVaRT, Motion Analysis Co.) was used for real-time motion capture and for post-processing and tracking of marker data. Three representative strides from five separate trials were used for analysis from each session. A Retest was performed with the same protocol at an interval of 4-weeks to evaluate the inter-session repeatability.

Kinematic data of foot segmental motion was collected and tracked using the Foot3D Multi-Segment Software (Motion Analysis Co., Santa Rosa. CA). The definition of the coordinate systems based on these markers and the method used to calculate the joint rotation and arch parameters had been described previously [8].

### Data analysis

We divided the gait cycle into 100 time points (1% interval between time points) for data analysis. Seventeen parameters were tested for repeatability. Hallux (flexion/extension, rotation), hindfoot (flexion/extension, pronation/supination, rotation), forefoot (flexion/extension, pronation/supination, rotation), medial forefoot (flexion/extension, pronation/supination, rotation), lateral forefoot (flexion/extension, pronation/supination, rotation) and arch parameters (arch height, arch length and arch index) were evaluated.

Coefficients of multiple correlation (CMC) and intra-class correlation coefficient (ICC) were analyzed in order to assess the intra-session repeatability. Intra-session CMC and ICC were only analyzed for the first sessions. Intra-session CMC was evaluated from the first 2 strides in the selected 3 strides of the first session. Intra-session ICC evaluations were based on the selected 3 strides.

For inter-session repeatability, CMC and ICC were analyzed. Data from the three trials for each visit were averaged, and then the inter-session CMC and ICC were evaluated.

The difference between the two sessions was assessed for each time points of the gait cycle. After that, the average, standard error, and range of the inter-session difference were calculated.

The range of motion (ROM) in each segmental foot motion was calculated in each participant. For the assessment of intra-session and inter-session repeatability of the ROM measurements, intra-session and inter-session ICC was analyzed. The intra-session ICC was evaluated based on the selected 3 strides of the first session and the inter-session ICC based on the average of each session.

We interpreted that 0.65 ≤ CMC (R) < 0.75 suggests moderate repeatability, 0.75 ≤ CMC (R) < 0.85 suggests good repeatability, 0.85 ≤ CMC (R) < 0.95 suggests very good repeatability, and CMC (R) ≥ 0.95 suggests excellent repeatability [19]. We interpreted that ICC < 0.5 suggests poor repeatability, 0.5 ≤ ICC < 0.75 suggests fair to good repeatability, and ICC ≥ 0.75 suggests excellent repeatability [35].

## Results

**Figure 2:**
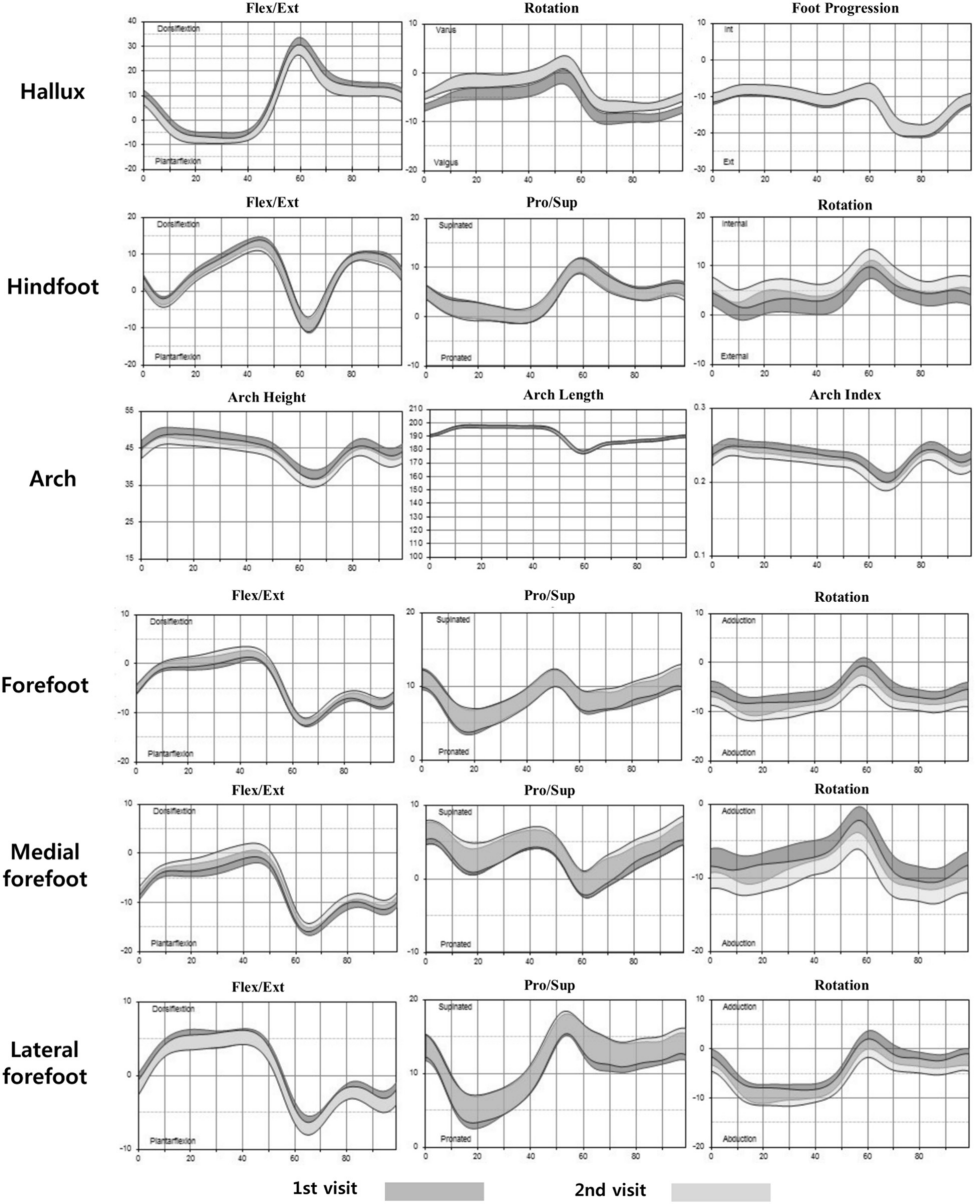
**Walking kinematics for the 1st and 2nd visit (average with a range representing 2 standard deviations).** Each row shows the motion of each segment: hallux, hindfoot, arch, forefoot, medial forefoot, lateral forefoot motion. Each column represents motion in each of the three planes (sagittal, coronal, transverse plane). Horizontal axis represents gait cycle, and vertical axis represents the range of motion.

The mean cadence was 113.5 (steps/min) and the mean speed was 124.1 (cm/sec). The mean stride length was 131.1 cm and the mean step width was 10.9 cm. Generally, the patterns of segmental movement seemed to be consistent. Inter-segment foot angles from healthy adults of a MFM with 15-marker set showed a narrow range of variability during the gait cycle (Figure 2).

The intra-session CMC by 1% interval of gait cycle is presented in Table 3. The mean intra-session CMC (±standard deviation) was 0.948 (±0.027). The intra-session CMC values of all parameters were interpreted as excellent or very good repeatability. The mean intra-session ICC was 0.981 (±0.010). The intra-session ICC values of all parameters were interpreted as excellent.

**Table 3:** Repeatability of foot kinematics

	**Intra-session CMC**				**Inter-session CMC**			
	**Present study**	**LFM **[10]	**MiFM **[28]	**HFM **[3]	**Present study**	**LFM **[10]	**MiFM **[28]	**HFM **[3]
Hallux								
Flex/Ext	0.971	0.987	0.974	0.993	0.796	0.851	0.950	0.984
Rotation	0.970	0.920	0.974	0.834	0.951	0.862	0.899	0.383
Hindfoot								
Flex/Ext	0.931	0.968	0.965	0.987	0.837	0.933	0.899	0.974
Pro/Sup	0.890	0.935	0.958	0.996	0.697	0.899	0.872	0.653
Rotation	0.927	0.907	0.945		0.728	0.854	0.892	
Arch								
Height	0.959				0.798			
Length	0.909				0.980			
Index^*^	0.952				0.729			
Forefoot								
Flex/Ext	0.978	0.928	0.907	0.985	0.840	0.741	0.732	0.675
Pro/Sup	0.993	0.924	0.946	0.974	0.687	0.801	0.881	0.919
Rotation	0.972	0.908	0.962	0.950	0.813	0.761	0.916	0.518

The CMC of intra-session and inter-session were calculated and compared with those from previous researches (Leardini foot model [10], Milwaukee foot model [28], and Heidelberg foot model [3]).

^*^Arch Index = Arch height/Arch length.

LFM, Leardini foot model; MiFM, Milwaukee foot model; HFM, Heidelberg foot model.

The inter-session CMC by 1% interval of gait cycle is presented in Table 3. The mean inter-session CMC was 0.801 (±0.077). The inter-session CMC values of hallux rotation and arch length were the highest, while those of hindfoot pronation/supination, hindfoot rotation, forefoot pronation/supination and arch index ranged between 0.687 and 0.729, being interpreted to have moderate repeatability. The mean inter-session ICC was 0.886 (±0.047). The inter-session ICC values of all parameters were interpreted as excellent. The inter-session ICC value of hallux rotation and arch index was the highest (0.974 and 0.989, respectively), whereas those of pronation/supination of the hindfoot (0.838) and forefoot (0.814) were the lowest.

The mean, standard error and range of inter-session differences at each time point (1% interval) is presented in Table 4. The inter-session difference of hindfoot flexion/extension and forefoot flexion/extension were lowest (1.3° and 2.6°, respectively), whereas those of hallux flexion/extension and hindfoot pronation/supination were the highest (4.0° and 4.3°, respectively).

**Table 4:** Repeatability of foot kinematics (the difference between two sessions)

	**Present study**			**OFM **[4]	**MiFM **[28]	**HFM **[3]
**Inter-session difference**	**Mean**	SE^*^	**Range**	**Mean**	**Mean**	**Mean**
Hallux (^o^)						
Flex/Ext	4.0	0.05	(0.17, 7.08)	6.0	3.63	1.97
Rotation	3.6	0.05	(0.28, 6.60)	6.4	2.78	1.45
Hindfoot (^o^)						
Flex/Ext	1.3	0.05	(0.31, 3.03)	1.4	2.37	1.34
Pro/Sup	4.3	0.05	(0.53, 6.19)	3.0	1.60	3.38
Rotation	3.0	0.04	(0.20, 5.94)	3.2	2.00	
Arch (mm)						
Height	4.9	0.06	(0.95, 8.31)			
Length	2.0	< 0.01	(0.00, 3.61)			
Index^**^	0.03	0.03	(0.01, 0.05)			
Forefoot (^o^)						
Flex/Ext	2.6	0.03	(0.16, 4.93)	2.9	2.54	3.93
Pro/Sup	3.0	0.03	(0.17, 6.09)	3.3	2.87	1.35
Rotation	3.9	0.04	(0.04, 7.19)	4.3	2.48	2.54

The mean inter-session difference of inter-segmental angle was calculated and compared with those from previous researches (Oxford foot model [4], Milwaukee foot model [28], and Heidelberg foot model [3]).

^*^Standard Error.

^**^Arch Index = Arch height/Arch length.

OFM, Oxford foot model; MiFM, Milwaukee foot model; HFM, Heidelberg foot model.

The range of motion in hallux flexion/extension and hindfoot flexion/extension were largest in all segmental motions, which were 39.7° and 22.2°, respectively. The mean intra-session ICC of ROM measurements in 17 segmental motion parameters was 0.992 (±0.004). Intra-session ICC values of ROM in all parameters were interpreted as excellent. The mean inter-session ICC was 0.940 (±0.028). Inter-session ICC values of ROM in all parameters were interpreted to have excellent repeatability.

## Discussion

We demonstrated that a MFM with a 15-marker set in absence of medial and lateral calcaneal markers had high intra-session and inter-session repeatability, which we believe can be applied to evaluation of inter-segmental foot motion during gait in healthy participants.

The positions and motions of the foot and ankle during gait are complex in the process of supporting the body, transferring forces from the ground and adapting to uneven surface [27]. A standard gait analysis which had been widely used for clinical decisions, especially in the treatment with neuromuscular disorders, was thought to be inappropriate to evaluate detailed motion of the foot segment because it considered the foot as a single rigid segment or a vector. Thus, several 3D MFMs have been introduced and evaluated in the last two decades, for in vivo analysis of dynamic foot kinematics [3-12]. In a recently published systemic review, Deschamps et al. [14] identified fifteen 3D MFMs qualified for evaluation and reproduction. They concluded that several published MFMs seemed to provide biomechanical parameters which can help clinicians in their decision making process and that the evidence for the continued use of 3D MFMs had been provided.

It is obvious that increasing number of markers with accurate placement will enable more precise analysis of actual segmental foot motion. However, placing multiple markers in a small area of interest might cause significant variability in marker placement. For example, in OFM, mean inter-session difference of hallux motion was highest (mean difference, 6.0°) among all parameters [4]. In MiFM, inter-session CMC of forefoot flexion/extension was as low as 0.732 [28].

The model that was used in this study was a MFM adding 6 markers placed on the foot in addition to Cleveland Clinic lower extremity marker set. This model involves ten markers placed on prominent anatomical points around the foot and ankle with notable absence of medial and lateral calcaneal markers. The prominent difference of this model from other previous foot models is that this model has no medial and lateral calcaneal markers and only one hallux marker, which is placed on the the middle of the hallux nail bed. In this model, the calcaneus’ medial-lateral axis is established by the cross product of the vector from the distal marker to the ankle joint and the vector from the distal marker to the heel marker. A single vector from the 1st metatarsal head to the dorsal hallux marker is analyzed for hallux motion. We used this model based on the fact that marker placement on the medial and lateral calcaneus can be problematic [9] although published models have been validated in healthy participants and in some pathologic conditions [10,36]. In many clinical situations, especially in patients with foot deformities or tendon disorders, the sustentaculum tali and peroneal tubercle are not easy to palpate. Although we still had concerns that hindfoot motion could be represented accurately without medial and lateral calcaneus markers, we hypothesized that this simple MFM without medial and lateral calcaneal markers can represent specific segmental motion of the foot in a reliable manner and tried to test its reliability. In this study, the temporal pattern of segmental foot motion during a gait cycle was similar to previous descriptions from other foot models, although the absolute number of measured angles was somewhat different. For example, the sagittal motion of hallux and hindfoot segments was quite similar with that of OFM, MiFM, and LFM. However, there were major differences in the description of coronal motion, especially in the forefoot segment when compared with other foot models.

When assessing the quantitative kinematic data of joint motion, measurement errors and variability are thought to come from three primary sources: (1) participant, (2) measurement system, and (3) examiner [32]. Natural variability exists even in the gait of “normal” participants, and can be partially attributed to many factors [37,38]. There is also some step-to-step variability within a participant. Natural variability of gait should be considered to be different from experimental error. Errors of the measurement system may include the accuracy of the motion analysis system used and skin motion artifact. Only a few published MFMs have been evaluated in terms of a system accuracy study [4,5,7,26,36]. However, variability from measurement system accuracy was consistent and attributed to very little portion of overall variability of the kinematic measurements [7,32]. Investigators have drawn a consensus that the majority of the variability in the kinematic measurement comes from the examiner [9,28,31,32,39]. Deschamps et al. [14] postulated in their systemic review that the systematic errors introduced by skin motion artifact and the difficulty of tracking specific bones in the foot seemed to be manageable elements. They commented that consideration should be given to between-day, between-trial, between-clinician (examiner), and between-participant repeatability.

In this study, we tried to evaluate intra-session (between-trial) repeatability, which is considered to reflect variability from gait variation within a participant and from the system measurement accuracy, and inter-session (between-day) repeatability, which can be considered to reflect the summation of all possible sources of error. We did not measure the between-examiner repeatability in this study. The between-examiner repeatability is considered to be mainly related with errors of marker placement. In several articles designed to evaluate between-examiner repeatability [9,16], all investigators reported that inter-session variability was greatly dependent on the experience of the examiners. We concur those conclusions from previous researches that between-examiner repeatability existed substantially regardless of MFMs and that it would depend on the experience of the examiners. Thus, we focused on the inter-session (between-day) repeatability to evaluate whether kinematic data in one participant can be estimated in a repeatable way using this simple model without medial and lateral calcaneal markers.

Considering between-participant variability, in most clinical laboratories, patient gait data is compared to the average response of “normal” control participants [39]. The control data, usually provided in ranges, supplies a reference for the study of pathological gait patterns. Our subsequent research on between-participant variability using this model has shown low variability in certain segmental motions of the foot in examinations of one hundred healthy participants (not published).

For the statistical analysis of repeatability, the CMC, which is the positive root of the adjusted coefficient of multiple determinations (CMD) has been used extensively to evaluate similarities between kinematic waveforms [14,28]. The ICC is also a widely used measure of waveform resemblance [14]. In this study, intra-session repeatability assessed by CMC and ICC could be interpreted to be excellent. The inter-session repeatability, which reflected a total summation of error in segmental foot motion measurements, could be interpreted to be excellent in all segmental foot motion parameters in the inter-session ICC analysis and good or very good in most parameters in the inter-session CMC analysis (Table 3). Considering that our researchers were not experienced in segmental foot modeling before this study, we thought our inter-session repeatability data was comparable with previous reports of other foot models. With accumulated experience of foot marker placement, the inter-session repeatability might be improved. However, we found that inter-session repeatability was lowest in the measurement of pronation/supination motion of hindfoot and forefoot segments, which might come from the intrinsic weakness of placing only two markers on the calcaneus and/or error of marker placement.

Some researchers, however, recommended including measures of absolute measurement error (e.g. SD and standard error of the mean), in order to fully appreciate reliability data [9,14,16]. We evaluated the difference between inter-session average values at each time point and found that the mean in inter-session difference ranged between 1.3° and 4.3°, which was comparable to previous reports in other foot models. The difference was rather consistent throughout all time points, which implied that the difference might come from the difference in marker placement. However, in clinical situations, the change of ROM in motion might be significant information to differentiate the abnormal condition from normal range. In this model, ROM measurements were quite consistent. The mean inter-session ICC was 0.940 (±0.028) and interpreted to be excellent in all segmental foot motion measurement.

Despite promising results for further utilization of a present MFM, limitations of this study may be addressed as follows. First, we are still concerned that defining the hindfoot anatomical coordinate system using just two posterior calcaneal markers and the ankle joint center in this system can be problematic. The single marker placement on the hallux also may be insufficient to represent three dimensional motion of the hallux. Whether this system can be applied to patients with foot deformity or disease should be clarified further. Secondarily, we did not assess between-examiner repeatability in this study. We agree that an experienced technician is a prerequisite to increase inter-session repeatability. However, because marker placement was performed by a single technician without previous experience of MFM, we could not assess inter-rater repeatability. Finally, we were not able to compare our results directly with the previously reported studies, because there are no previous reports on the reliability of the present model.

## Conclusion

In this study, we used a MFM with 15-marker set in order to evaluate segmental foot motion in healthy adults. We demonstrated that this model had high intra-session and inter-session repeatability in the assessment of foot motion. We believe that this 3D MFM can be applicable in clinical settings, which require further elucidation.

## Competing interests

The authors declare that they have no competing interests.

## Authors’ contributions

SGS, DYL and IHC conceived of the study; participated in design of the study; performed the data analysis including statistical analysis; and participated in drafting the manuscript. HJM, JHK, KML and CYC participated in its design and coordination and helped to draft the manuscript. SJK helped to perform statistical analysis. All authors read and approved the final manuscript.
